# Consistent Robustness Analysis (CRA) Identifies Biologically Relevant Properties of Regulatory Network Models

**DOI:** 10.1371/journal.pone.0015589

**Published:** 2010-12-16

**Authors:** Treenut Saithong, Kevin J. Painter, Andrew J. Millar

**Affiliations:** 1 Department of Biological Sciences, Institute of Molecular Plant Sciences, University of Edinburgh, Edinburgh, United Kingdom; 2 Department of Mathematics and Maxwell Institute for Mathematical Sciences, School of Mathematical and Computer Sciences, Heriot-Watt University, Edinburgh, United Kingdom; 3 Centre for Systems Biology at Edinburgh, University of Edinburgh, Edinburgh, United Kingdom; 4 School of Bioresources and Technology, King Mongkut's University of Technology Thonburi, Bangkok, Thailand; Centre for Genomic Regulation (CRG), Universitat Pompeu Fabra, Spain

## Abstract

**Background:**

A number of studies have previously demonstrated that “goodness of fit” is insufficient in reliably classifying the credibility of a biological model. Robustness and/or sensitivity analysis is commonly employed as a secondary method for evaluating the suitability of a particular model. The results of such analyses invariably depend on the particular parameter set tested, yet many parameter values for biological models are uncertain.

**Results:**

Here, we propose a novel robustness analysis that aims to determine the “common robustness” of the model with multiple, biologically plausible parameter sets, rather than the local robustness for a particular parameter set. Our method is applied to two published models of the *Arabidopsis* circadian clock (the one-loop [1] and two-loop [2] models). The results reinforce current findings suggesting the greater reliability of the two-loop model and pinpoint the crucial role of TOC1 in the circadian network.

**Conclusions:**

Consistent Robustness Analysis can indicate both the relative plausibility of different models and also the critical components and processes controlling each model.

## Introduction

Mathematical modelling has established itself as a complementary means to study the complexity of biological systems. Through its capacity to integrate extensive data from diverse sources [3]-[5], modelling has contributed greatly to our understanding of the mechanisms governing organismal behaviour [1], [2], [6]–[10], as exemplified by the JWS online (http://jjj.biochem.sun.ac.za/) [11] and BioModels (http://www.ebi.ac.uk/biomodels-main/) [12] databases.

The fitting of models to data necessitates the determination of parameters describing processes of the biological system [13]–[15]. However, parameters obtained through experimental measurement are condition-dependent, while the measuring process itself is costly with respect to technique, expense, and time. Optimisation provides an alternative and increasingly popular method to estimate the model parameters [16]. Implementing the optimisation requires an appropriate measure to compare the experimental data with simulated results and the first test of a model's suitability lies in its capacity to “fit” the biological data. However, a considerable drawback in using optimisation to estimate parameters for complex models is that multiple parameter sets may “fit” the data equally [1], [17].

An analysis of the robustness of the system is the logical next step to address the uncertainties arising from considering only “goodness of fit”. While the notion of model robustness is interpreted broadly in the literature, the robustness of a biological system is mainly defined as a property of a biological function [15], [18]. Measurement of the robustness of a biological system therefore relates to the determination of the effect of certain perturbations on the biological function. In this context, the biological function is inferred by “the behaviour of a dynamical system”- such as a gene expression waveform or the period of a sustained oscillation. These behaviours could be among the targets used in the optimisation process. Hence, the reference to model robustness here is specifically defined as the persistence of the model behaviour against perturbations, as reflected in the deviations of simulations from biological data. The results of robustness analysis can be used as outlined, for example, in Morohashi *et al* (2002) [19], where it is suggested that robustness should be an essential property for any biological system and can therefore be considered as a decisive factor for selecting a credible model or pinpointing the weaknesses of a failed model. Bifurcation analysis applied to two published models for the *Xenopus* cell cycle oscillator [20], [21] indicated that the later model is more robust, thus cementing its position as the more realistic model than based on biological evidence alone. In a similar manner, Zeilinger *et al* (2006) [17] demonstrated that three distinct models for the Arabidopsis circadian clock could be distinguished through robustness analysis.

Robustness/sensitivity analysis can also be used to pinpoint the specific factors or processes affecting a system, indicating how the system maintains functionality in spite of internal or environmental perturbations [22], [23]. Furthermore, robustness analysis reveals insight into the importance of model parameters on the model behaviours [24]. A variety of techniques have been developed to determine the robustness of a system, for example bifurcation analysis [25]–[27], control analysis (CA) [28]–[31] and Infinitesimal Response Curve (IRC) [32]. To summarise such analyses and compare across the systems, Kitano (2007) [33] proposed a method to quantify the robustness through a single factor. The above methods reveal different insights into the robustness of distinct system properties, for example bifurcation analysis can determine the exact space of the parameters giving desired system performance (*e.g.* periodic solution for oscillator) [25]–[27], while CA and IRC can quantify the dynamic changes of the system in applied differentiated perturbations [9], [34]–[36]. Although CA and IRC provide precise analytical measurements, these methods evaluate the robustness around a fixed point in parameter space and the subsequent results are therefore potentially biased to a specific parameter set. The inherent impact of parameters to model robustness is hard to separate [13]–[15] and it becomes exaggerated in mechanistic modelling, where the focus is on correct interactions rather than the used parameters.

The circadian clock is a fundamental biological process of organisms ranging from unicellular (*e.g. Synechococcus* cyanobacterium) to multi-cellular [37]–[39]. Its network is believed to be composed of a negative feedback loop structure which generates a robust 24h-period oscillation. While the molecular mechanism of the circadian clock has been extensively studied in the cyanobacterium [37], [38], [40], fungi (*Neurospora crassa*) [37], [38], [41], [42], insects (*Drosophila melanogaster*) [37], [38], [43] and mouse [37], [39], for plants (*Arabidopsis thaliana*) the network has recently been established [38], [44]–[46]. A series of Arabidopsis circadian clock models were constructed following the proposal of its molecular network. Locke *et al* (2005) [1] created an initial “one-loop” model based on the hypothesis of Alabadi *et al* (2001) [44], which proposed a negative feedback loop of three genes (Figure S1a): two redundant gene encoding MYB transcription factors, *LATE ELONGATED HYPOCOTYL* (*LHY*) and *CIRCADIAN CLOCK ASSOCIATED 1* (*CCA1*), and a gene encoding the pseudo-response regulator protein, *TIMING OF CAB EXPRESSION 1* (*TOC1*). A system of seven ordinary differential equations (ODEs) containing 25 parameters was proposed to describe the regulation of the circadian clock for the one-loop model. While the simulated results of this model fitted experimental data from the wild-type (see Figure S1a), it failed to match mutant data, for example short period oscillations observed in the *lhy;cca1* double mutant plant [1], [2]. To match these data, Locke *et al* (2005b) [2] derived a second model (the two-loop model) through addition of hypothetical genes ‘*X*’ and ‘*Y*’. The hypothetical gene ‘*X*’ was added to extend the time-delay in the model and incorporate an indirect activation of *LHY/CCA1* by *TOC1* (whose mechanism is unclear) [44], [47]. An additional loop connects with *TOC1* in the original loop in an interlocking fashion as illustrated in Figure S1b. The extensions resulted in a system of 13 ODEs and 58 parameters. Simulations of the two-loop model match additional experimental data, including the *lhy;cca1* double mutant.

Parameter optimisation to fit such data can reveal multiple parameter sets spanning large tracts of parameter space. Until the parameters are measured experimentally, it is desirable to determine the sensitivity/robustness of a model circuit *independent* of the chosen parameter set and here we propose a strategy that determines this *intrinsic* robustness of a model. The method is applied to the one- and two-loop models for the *Arabidopsis* circadian clock, where we take advantage of the previously globally-optimised parameter sets produced by Locke *et al* (unpublished data) as an initial input for the method. We demonstrate that robustness corroborates the perceived greater credibility of the two-loop model, which is more robust, as well as matching more data than the one-loop model. Our analysis leads to biological inference on the core processes governing this network.

## Results

### Analysis of Arabidopsis circadian clock models

The proposed method, Consistent Robustness Analysis (CRA), was applied to analyse two published models of the Arabidopsis circadian clock. The circadian clock in Arabidopsis is appropriate for a number of reasons. Firstly, circadian clocks are believed to be highly robust in comparison to other cellular processes (for example, calcium or glycolytic oscillations) [48]. Secondly, previous studies have demonstrated that the one-loop model failed to capture a critical behaviour of the Arabidopsis circadian clock that was replicated in the two-loop model. A critical test of the procedure introduced here is to determine whether it can extend understanding beyond the better fit of the two-loop model. We describe the analysis of the two-loop model in detail, summarise the main results from a similar analysis of the one loop model and interpret the results biologically.

### 1. Robustness analysis of the two-loop Arabidopsis circadian clock model

#### (1) Reference parameter set selection

**Figure 1:**
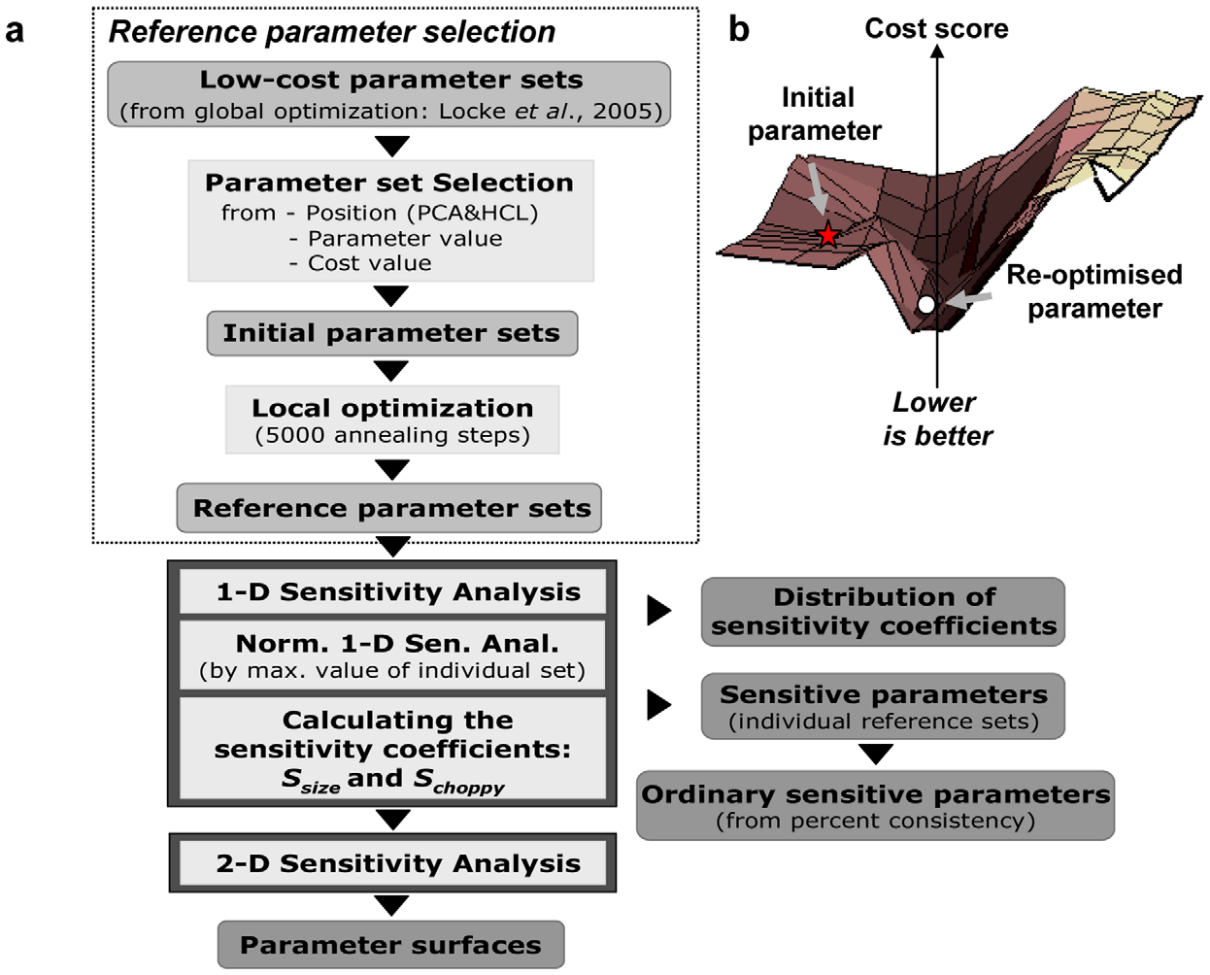
Consistent robustness analysis scheme. (a) Schematic demonstrating the proposed consistent robustness analysis method which aims to acquire the universal robustness property of a model producing similar results in wide regions of reasonable parameter space, illustrated in (b).

The input to our analysis exploits earlier work by Locke *et al* (unpublished data), in which 50 low cost-of-fit parameter sets were generated following global optimisation to the semi-quantitative cost function (see [1] for details). One of these, set 0, was described previously [2]. A set of reference parameter sets was selected as described in Methods (see also Figure 1a). Hierarchical Clustering (HCL) and Principal Component Analysis (PCA) were employed to measure the distances between the parameter sets. Figure S2a plots the HCL results for the full 50 parameter sets and the asterisks mark those selected for the second phase of the analysis. We note that, among the seven selected parameter sets, four (*sets 9*, *12*, *13*, and *14*) are distant from *set 0*, while the others (*sets 27* and *39*) are located close to *set 0*. Re-optimisation of each of the selected parameter sets to the fully-quantitative cost function (fitting to data) was performed through a simulated annealing algorithm (5000 steps) to yield the locally optimised parameter sets, termed *L0*, *L9*, *L12*, *L13*, *L14*, *L27* and *L39*, to be used in the later sensitivity analysis. The simulations given from *L0* match the data very well (Figure S1b) compared to those from the rest whose simulated oscillations showed low amplitude (*L12* and *L13*; Figure S3), abnormal shape (*L12* and *L27*; Figure S3), and short period in continuous darkness (all of parameter sets; Figure S3). The unequal fit quality of the selected parameter sets indicated that the re-optimisation to quantitative cost function is required to refine the initial results obtained from exhaustive search against the semi-quantitative criteria of Locke *et al* (unpublished data). To illustrate the span of the selected parameter sets used in the analysis, the re-optimised parameter values are plotted in Figure S2b.

#### (2) One-dimensional sensitivity analysis

**Figure 2:**
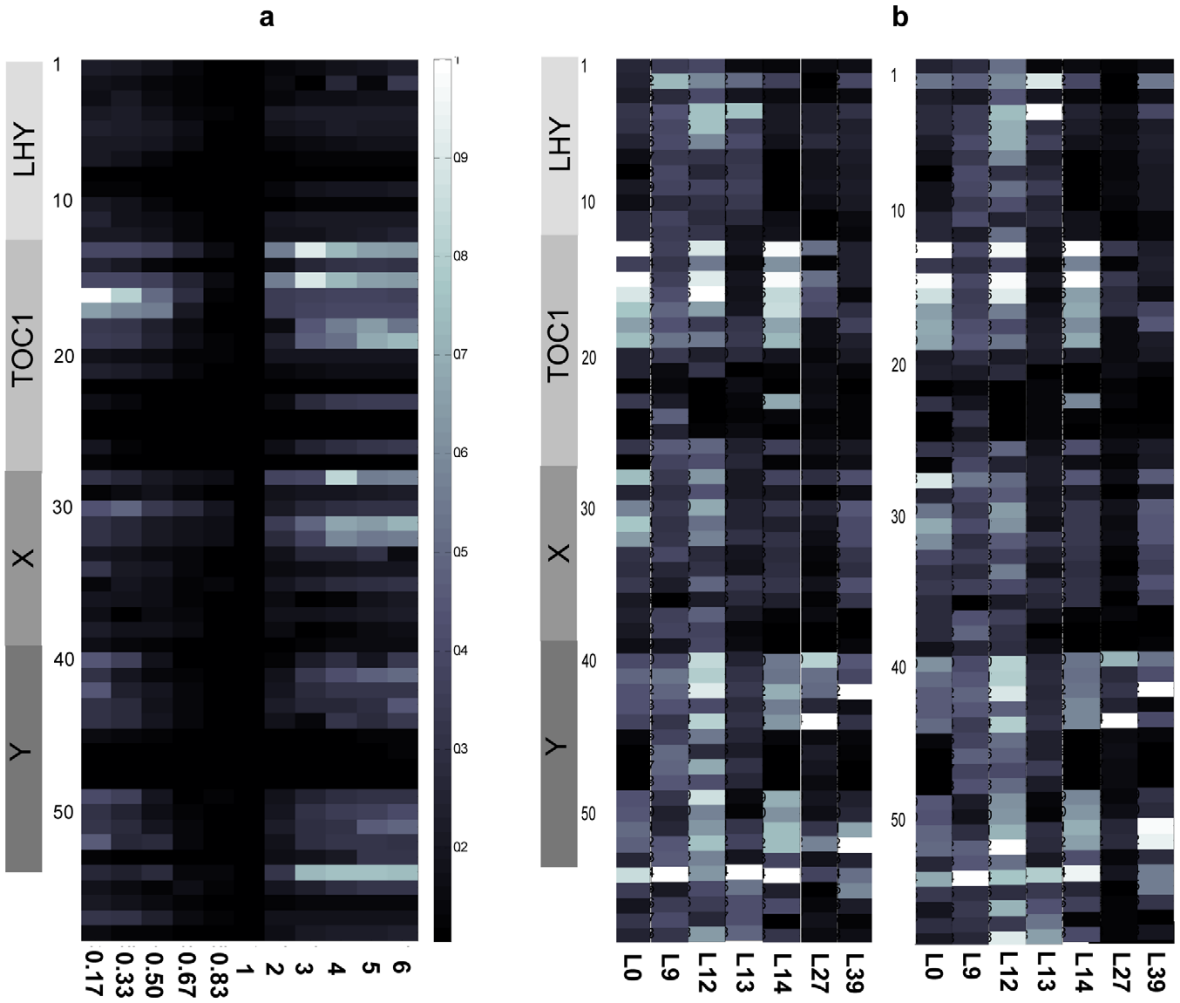
Sensitivity of the two-loop model of Arabidopsis circadian clock. The (a) sensitivity with respect to the parameters in the two-loop Arabidopsis circadian clock model using *L0* (the reoptimised parameter set from *set 0*). The heatmap plots the sensitivity (white  =  sensitive, black  =  robust) of the model at all parameters (rows) and perturbations (columns). Similar plots for other reference parameter set shown in Figure S4 for sensitivity. (b) The sensitivity coefficients (*S^size^* - left panel and *S^choppy^* – right panel) of the two-loop model for all reference parameter sets are plotted as a heatmap in which high sensitivity is shown in white, scaling to low sensitivities in black. The sensitivity coefficients of a parameter (row) in each reference parameter set (column) were independently determined from the cost function normalised within the reference set. Note that *S^size^* and *S^choppy^* (Figure S5) are broadly consistent, indicating that either method is reasonable.

**Figure 3:**
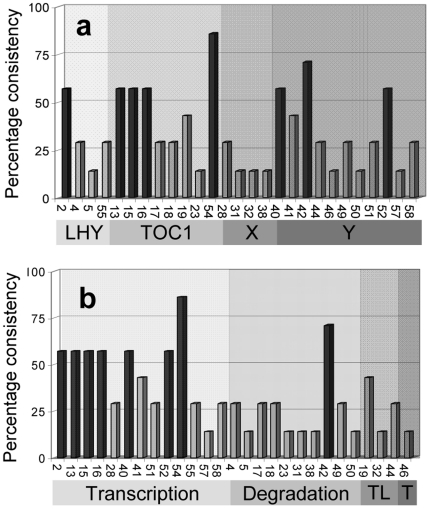
Percent consistency of sensitive parameters in the two-loop Arabidopsis circadian clock model. The percent consistency of the sensitive parameters (using *m*  = 1) among the reference parameter sets was plotted according to (a) the genes and (b) molecular processes (TL =  translation and T  =  Transportation). The consistently sensitive parameters, marked by black bars, were classified based on a 50 percent consistency cut-off.

One-dimensional analysis was performed through six-fold (plus and minus) perturbations of each parameter in each of the reference sets. Figure 2a displays the sensitivity for the two-loop model for *L0*: results for each of the other parameter sets can be found in Figure S4. Sensitivity/robustness is determined first by calculating the change to the cost-of-fit due to each parameter perturbation and then normalising within the parameter set according to Equation (2). The sensitivity coefficients are determined from Equations (3-4) and summarised in Figure 2b (scales from highest in white to lowest in black). The normalisation is required because the unperturbed cost-of-fit and the maximum perturbed cost-of-fit are different for each parameter set. This means that absolute values for sensitivity coefficients can only be compared within a column (*i.e*. across the parameters within a particular parameter set). Nevertheless, there are similar trends with respect to the sensitivity of a particular parameter across the different sets. Distinct sets of sensitive parameters are calculated for each parameter set (according to the classification criterion in Equation (5) with *m*  =  1) and listed in Table 1. Between five and thirteen sensitive parameters were determined for each set, resulting in a pool of 27/58 parameters being identified as sensitive at least once (Table 1). The discrepancy in which parameters are identified as sensitive for the various parameter sets highlights the fact that model sensitivity depends on the parameter set as well as the network circuit. On the other hand, certain parameters were repeatedly classified as sensitive across the diverse parameter sets. The frequency with which the parameters are identified as sensitive is tabulated in Figure 3 (see also Figure S5) and, stipulating that a particular parameter must be identified as sensitive in at least 50% of sets, we determine the eight “consistently-sensitive” parameters listed in the final column of Table 1. These eight consistently sensitive parameters are P2 (*n1*: max. light-dependent *LHY* transcription), P13 (*n2*: max. *TOC1* transcription rate), P15 (*n3*: constant of *LHY* inhibiting *TOC1* transcription), P16 (*g3*: constant for *TOC1* transcription), P40 (*n6*: constant for *Y* transcription), P42 (*m12*: max. degradation rate of *Y* mRNA), P52 (*g6*: constant for *Y* transcription), and P54 (*b*: Hill coefficient for *TOC1* transcription) (see also in Data S1). Since the classification of a sensitive parameters is subject to the strictness of the classification criterion (*m* in Equation (5); see also Figures S5-S6), we varied *m* to test the appropriateness of the employed value. For *m*  =  0.5 (Figure S6a) or *m*  =  2 (Figure S6b), we obtained respectively too many and too few sensitive parameters, suggesting that the applied criterion of *m*  =  1 is relatively more sensible.

**Table 1:** Summary of the sensitivity analysis of the one-loop and two-loop Arabidopsis circadian clock models.

One-loop Arabidopsis Circadian clock model (25 parameters)									
Description	*L2*	*L26*	*L31*	*L32*	*L37*	*L41*	*L50*	Pool of SP	CSP
Number of SP	4	2	3	7	4	3	5	7	3
SP	*n2* *g2* *m4* *k4*	*m4* *k4*	*n2* *g2* *m4*	*n2* *g2* *m4, m5, m6* *k4* *p2*	*n2* *g2* *m4, m5*	*n2* *g2* *m4*	*n2* *g2* *m4, m5* *k4*	*n2* *g2* *m4, m5, m6* *k4* *p2*	*n2* *g2* *m4*

Two-loop Arabidopsis Circadian clock model (58 parameters)									
Description	*L0*	*L9*	*L12*	*L13*	*L14*	*L27*	*L39*	Pool of SP	CSP
Number of SP	11	8	13	6	13	9	5	27	8
SP	*n2, n3, n4, n6* *g3* *m4* *k4, k7* *p2, p3* *b*	*n1, n4* *g5* *m12* *k9* *p2* *r8* *b*	*n2, n3, n6* *g3, g5, g6* *m1, m12, m14* *k1* *p4* *b, d*	*n1* *m1* *a, b, d, e*	*n2, n3, n7* *g3, g6* *m4, m6, m12,m14* *k4, k12* *p2* *b*	*n1, n2,n3, n6, n7* *g3, g5* *m12* *p4*	*n1, n6,* *n7* *g6* *m12* *a, b*	*n1, n2,n3, n4, n6, n7* *g3, g5, g6* *m1, m4, m6, m12, m14* *k1, k4, k7, k9, k12* *p2, p3, p4* *r8* *a, b, d, e*	*n1, n2, n3,n6* *g3, g6* *m12* *b*

*SP  =  sensitive parameter, CSP  =  consistently sensitive parameterSupporting Information Legends

The sensitivity analysis pinpoints the influence of specific molecular processes, entities or parts of the genetic network: 7/8 of the consistently sensitive parameters describe transcription processes, 4/8 relate to the *TOC1* gene while 7/8 relate to evening-phase genes, *TOC1* and *Y*. Ranking the sensitivity coefficients, the most sensitive parameters correspond to *TOC1* transcription.

**Figure 4:**
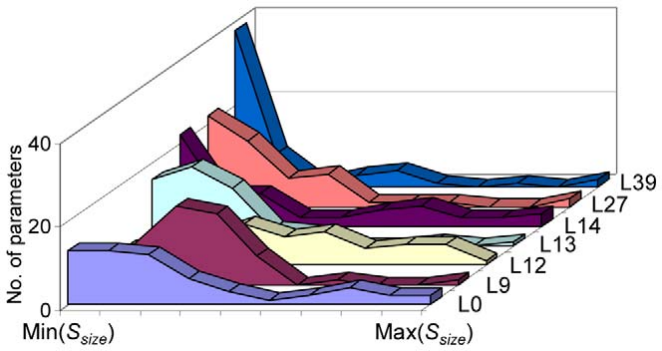
Distribution of sensitivity coefficients of parameters in two-loop Arabidopsis circadian clock model. The histograms demonstrate the distribution of the sensitivity coefficient (*S^size^*) within each reference parameter set of the two-loop model. This shows the frequency distribution of parameters of the model displaying similar magnitudes of sensitivity.

Further insight arises through the distribution of the parameter sensitivities. The distribution curves for sensitivity coefficient (*S^size^*) are plotted for the individual reference sets and presented in Figure 4. Comparing with Figure S2a, parameter sets located close to each other in parameter space show similar parameter sensitivity distributions, *e.g.* L0, L27, and L39 show a comparable pattern of the distribution curve (left-skewed with a small divided peak) while others demonstrate a seemingly random shape. The distribution of parameter sensitivity probably therefore reflects the relative position of the parameter set in parameter space. In the reverse direction, the distribution of parameter sensitivity for a new parameter set might be predicted from knowing its relative location within the parameter space.

#### (3) Two-dimensional sensitivity analysis

**Figure 5:**
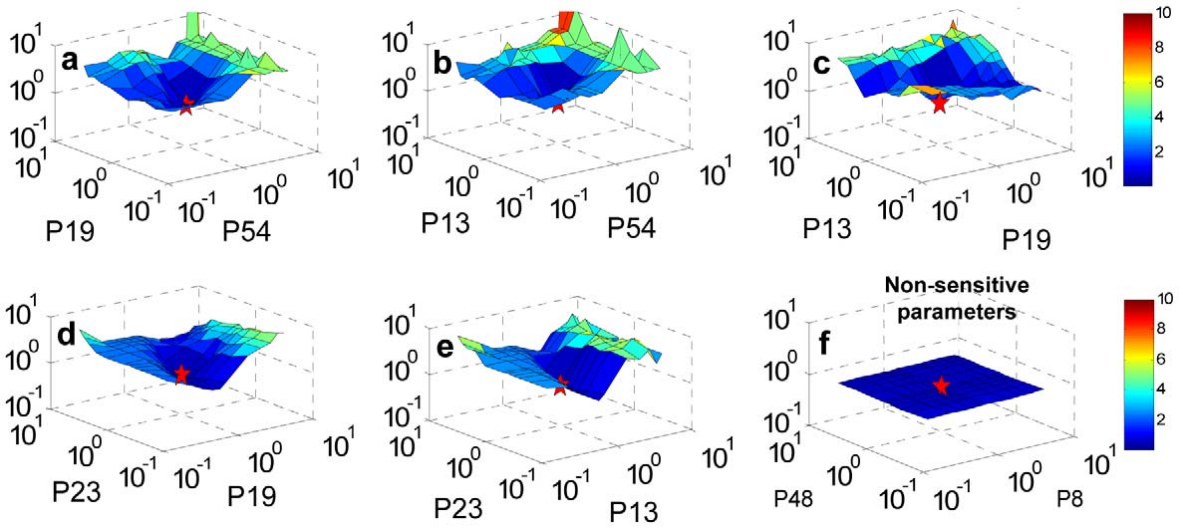
Two-dimensional sensitivity analysis of the two-loop Arabidopsis circadian clock model. The parameter surface obtained from a 2D sensitivity analysis of the two-loop model according to the (a–e) highly and (f) lowly sensitive parameters in set *L0*: P8 (*r2*)-TOC1 protein transportation to cytosol, P13 (*n2*)-max. *TOC1* transcription rate, P19 (*p2*)-rate constant of *TOC1* mRNA translation, P23 (*m6*)-max. rate of light independent cytoplasmic TOC1 degradation, P48 (*k11*)-Y protein in cytosol degradation, and P54 (*b*)-Hill coefficient of activation by protein Y. The red star illustrates the position of the reference parameter set which is always coincident with the minimum cost on the parameter surface. X and Y axis represent the perturbation of sensitive parameters while Z axis is the cost function corresponding to the parameter perturbation.

Greater insight into the robustness of the parameter space is obtained through two-dimensional sensitivity analysis. The long numerical time required to perturb across two dimensions in parameter space prevents an exhaustive analysis: the focus here is therefore on the most sensitive parameters as revealed in Step 2 (Figure 1). Insensitive parameters are expected to give rise to flat and smooth distributions (for example, see Figure 5f). The characteristics of the parameter surface can be inferred through limited investigations in “meaningful areas” of the most sensitive parameter space. A pair of highly sensitive parameters within a set were chosen and perturbed pair-wise. Examples of the parameter space surface of set L0 are plotted in Figures 5a–5e: the surface in highly sensitive regions is coarse, with a deep hole corresponding to where the optimal solution (red star) lies. Similar results for the other six reference sets are illustrated in Figure S7.

The parameter surface is an atlas of model sensitivity on the parameter coordinates, its nature demonstrating the range of model behaviours tested by the cost function at any given parameter set. Besides the main information, the efficiency of the optimisation procedure is also illustrated in these 3D maps, where the reference parameter sets were always located at the lowest point of the surfaces.

### 2. Robustness analysis of the one-loop Arabidopsis circadian clock model

A similar analysis was performed for the one-loop model and we state the main results for brevity. Again, the initiating globally-optimised parameter sets are provided by Locked *et al* (unpublished data). In contrast to the two-loop model, the robustness analysis demonstrated that the model is extremely sensitive to a specific minor group of parameters, which are generally conserved across all reference parameter sets. Overall, 7/25 sensitive parameters were identified, all of which relate to molecular processes of *TOC1* (transcription, translation, transportation and degradation). With the same consistency cut off (50%), three consistently sensitive parameters were defined as followed: P13 (n2: max. *TOC1* transcription rate), P14 (g2: Constant of activation by TOC1), and P15 (m4: constant Max. rate of *TOC1* mRNA degradation). The results indicate that *TOC1* transcription is the crucial process within the one-loop model. The sensitivity to *TOC1* in both the one-loop and two-loop models highlights its importance at the heart of the Arabidopsis circadian clock network.

### 3. Robustness analysis and Model plausibility

**Figure 6:**
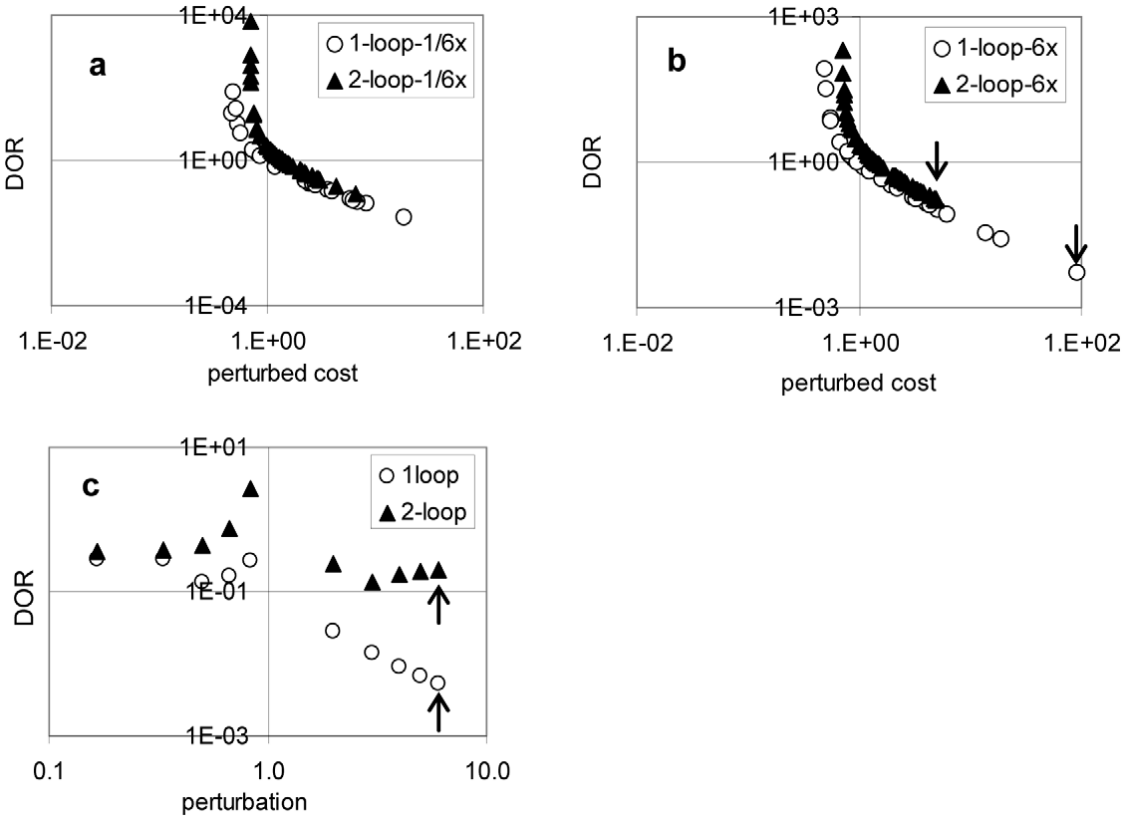
Comparison of model robustness. The robustness of the one-loop and two-loop Arabidopsis circadian clock models is compared through their best-fitting parameter set (*L0* for two-loop and *L26* for one-loop). The degree of robustness (*DOR*) of all parameters in both models at (a) 1/6 times perturbation, and (b) 6 times perturbation is plotted against the perturbed costs. The *DOR* of the most sensitive parameter in both models, as pointed out by the arrow in (b), was selected to plot across its full perturbation range in (c).

The two Arabidopsis clock models both express similar patterns with respect to sensitivity of the specific molecular components/processes, yet the degree of their sensitivity diverges. The robustness of the two models was compared through the *DOR* according to Equation (7). Figures 6a and 6b compare robustness between the one-loop and the two-loop models across all parameters at the largest perturbations. Robustness of the most sensitive parameters in each model (suggested by Figure 2b and marked by an arrow in Figure 6b), is graphed across its full perturbation range in Figure 6c. The robustness difference between the one-loop and two-loop models (determined through the most sensitive parameter pointed by arrows in Figure6c) demonstrates that the two-loop model is far more robust than the one-loop model for all parameters and across the perturbation range. Robustness can be considered as an essential property for most biological systems (particularly circadian clocks) and our analysis indicates the two-loop model is much more plausible as a model for the Arabidopsis circadian clock, reinforcing similar suggestions based on biological evidence [45], [47], [49]–[51]. Furthermore, it indicates that the analytical process developed here gives a reasonable measure for determining the robustness of the system, rather than its robustness at a particular point in parameter space.

## Discussion

Simple robustness analyses have limited relevance in systems biology. The measured robustness of a model to local parameter changes can vary according to the starting parameter set, exemplified by the distinct sets of sensitive parameters (SP) identified for each reference parameter set (Table 1), and in most biological systems only a minority of parameter values have been fixed by experimental measurements. “Global” analysis methods avoid this limitation by testing many starting parameter sets, to derive broader conclusions about the circuit of the model rather than the particular dynamics of one parameter set. These are often the most relevant to guide experimental work, because molecular and genetic studies commonly manipulate the model circuit rather than modulating parameter values.

The Consistent Robustness Analysis (CRA) developed here aims to identify a set of consistently sensitive parameters, for a range of biologically-reasonable parameter sets, which we term reference parameter sets. The method is more strategic than previous robustness analysis [52], as it focuses on parameter sets that best allow the model to match a full set of training data, avoiding time-consuming sensitivity analysis of parameter sets that cannot describe the biology of interest. It is still computationally costly, because multiple parameter sets that match the data must first be identified [1], [2]. Parameter sets that represented different dynamics (different parts of parameter space) were then manually selected, though this could in principle be automated.

The CRA approach has identified a subset of parameters for the Arabidopsis clock models that prove to be consistently sensitive for multiple parameter sets. For the two-loop model, only eight consistently sensitive parameter (CSP), mostly involved in *TOC1* transcription, were identified from an overall pool of 27 locally sensitive parameters (SP), suggesting wide variation between the sets of sensitive parameters (or genes/molecular processes) classified from each reference parameter set (Table 1). These “consistently sensitive parameters” (see Table 1) suggested two features: (1) the importance of *TOC1* transcriptional regulation in both models, as the parameters involved were always more than half of the whole set of sensitive parameters, and (2) the importance of the evening feedback loop involving *TOC1* and *Y* in the two-loop model, compared to the loop involving *LHY/CCA1* and *TOC1*, as the majority of consistently sensitive parameters related to *TOC1* or *Y* function, compared to only few of them (<15%) relating to *LHY/CCA1* or *X*. These traces are consistent with the results of an independent study of the two-loop model, which also inferred the dominance of *TOC1* transcription in controlling the model behaviours and properties [16].

In our relatively simple models, these results can be understood relatively easily, as follows:

Multiple experimental results support the importance of *TOC1* for circadian clock function. Manipulating *TOC1*, by loss-of-function mutants and transgenic over-expression or constitutive expression, severely alters circadian period and phase [43], [46], [48], [49] or may lead to arrhythmia [53]. Reflecting this importance, *TOC1* RNA and proteins are the components that interlock the feedback loops of the two-loop model. The range of available data may be biased, however, because *TOC1* was the earliest clock mutant described in Arabidopsis [54].

The relative importance of the evening loop in the two-loop model may be related to rhythm generation or to the input of light signals that regulate clock components. The two-loop model was constructed to account for the short-period rhythms of *lhy, cca1* double null mutant plants [2]. Accordingly, the evening feedback loop between *TOC1* and *Y* was required to sustain short-period rhythms in the model in a simulated *lhy, cca1* double null mutant: the model is relatively robust to the abolition of *LHY/CCA1* function. No such constraint was placed upon the simulated *Y* null mutant, which becomes arrhythmic [2]. In the later, three-loop model [8] the *Y* null mutant remains rhythmic. Robustness analysis of the three-loop model might be expected to show greater robustness to parameter changes in the evening feedback loop, in contrast to the sensitivity of this loop in the two-loop model.

Many of the data sets used in our analysis reflect regulation under constant light or in light:dark cycles, where the lights-on and lights-off signals at dawn and dusk both participate in entraining the Arabidopsis clock [55], [56]. In the two-loop model, these signals are mediated by the light-activated transcription of *LHY/CCA1* and of *Y*, respectively. The importance of the *TOC1-Y* loop in our results is consistent with simulations of the two-loop model under different photoperiods, where entrainment by the *Y-*mediated lights-off signal dominated the *LHY/CCA1*-mediated lights-on signal [8].

Finally, parameters related to transcription were extremely influential in both models. While the impact of transcription on the circadian rhythms in plants is unclear, an experimental study for the mammalian circadian clock has been undertaken by Dibner *et al*, demonstrating that reduction in global transcriptional rates resulted in resilient expression of core clock genes, for instance short rhythmic period and low amplitude [57]. Post-translational regulation is represented much less in the models than in current data on the clock mechanisms of several organisms [58]–[60]. The data available to construct these models, in contrast, strongly emphasised transcriptional regulation. Our results highlight the locations in the model where this emphasis should be revisited and confirmed experimentally: in the processes relating to the consistently sensitive *TOC1* transcriptional parameters, for example, whereas there is less evidence from our analysis that modelling of *LHY/CCA1* transcription needs to be revisited.

The plausibility of models can be impartially distinguished through comparing model-specific robustness using the parameter-independent robustness analysis (CRA) proposed in this work. While we acknowledge that robustness has been variously defined in the literature, the employment of the *DOR* definition here is a convenient and simple mathematical measure to quantify changes in model behaviours and compare differences between models. We further note the plausibility of this particular definition is strengthened by a number of recent publications using a similar measurement [16], [52]. While neither *DOR*, as defined in Equation 7, nor CRA can exclude the effect of redundancy (as described in [61]) from the robustness, this factor is still usable as a means to contrast robustness in diverse models: the redundancy effect is trivial in a small genetic network model and can be avoided in larger models by confining the degree of perturbation to a relatively small range with respect to the null mutation.

The comparison of the *DOR* both at the most sensitive parameter (Figure 6c) and across a full range of parameters within a parameter set (Figures 6a and 6b) suggests the greater plausibility of the two-loop model of Arabidopsis circadian clock, correlating with the previous assertion that the one-loop model contains a number of weak points. Circadian clock systems, in particular, require a degree of sensitivity to external environmental signals, *e.g.* light, for entrainment purposes, but should be highly robust to the internal (parameters) variations, as found in the more plausible two-loop model. The accuracy in determining model robustness here is expected to increase with the number of analysed reference sets, however in practice this is confined by the solutions of the optimisation.

As the CRA method has provided reasonable results for these relatively simple models, it is likely to provide greater advantages in analysing the larger models of more complex biological regulators, including plant clock models that include additional components known from the literature [45].

### Conclusions

Recently, robustness has been proposed a validating property of biological models: a reliable model should be highly robust. The analytical approach to characterising the real robustness of a model is therefore of the utmost importance. Herein, we created a new robustness analysis method called ‘consistent robustness analysis’ which intends to evaluate model robustness independently of operating parameters. This novel method allows us new comprehension into the given model: (1) the sensitive parameters of the model at a given parameter set, (2) the “consistently sensitive parameters” specific to the model, (3) the distribution of parameter sensitivity within the model, and (4) the parameter surface. In addition, we initiated a benchmark factor, (*DOR* or *DOS*), to evaluate the plausibility of various models (of differing complexity) by comparing the normalised magnitude of the model robustness. The success of this new method was demonstrated through the study of two Arabidopsis circadian clock models (one-loop and two-loop) with its results conferring both physically and biologically reasonable outcomes. The consistently sensitive parameters successfully pinpointed the *TOC1* transcription as the sensitive component and the molecular processes controlling the model behaviours, whereas *DOR* indicated the much greater plausibility of the two-loop model compared with the one-loop model, supporting many biological findings.

## Methods

### Modelling through fitting to the data

The optimisation process identifies parameter sets that minimise an appropriate *cost function:* a set of criteria or desired properties that a “good model” should satisfy. The *cost function* typically compares or quantifies the mismatch between the behaviours of the model and the real system, for example experimental data sets and/or qualitative criteria from observed biological behaviour [1]. In the analysis performed in Results section, the cost function compares simulated results with experimental data from various sources/conditions (see also Table S3 in Data S2) [44], [50], [62], [63] using a least-square formula [64]. A low cost-of-fit is thus expected to give a good representation of the system. However, for the large parameter spaces typical of complex models, it is unlikely that a unique minimising parameter set exists and similar fitting results may be obtained from widely spaced parts of the parameter space. Furthermore, experimental data is collected under various conditions in different laboratories, thus altering specific parameters. Consequently, the extent to which robustness of a model can validly be determined from a single parameter set is uncertain.

### Consistent Robustness Analysis (CRA)

We propose a new analysis to address some of the limitations highlighted above. The aim is to understand system robustness by performing sensitivity analyses using multiple parameter sets that yield reasonable model behaviour, as judged by the full cost function. Figure 1a illustrates our algorithm, consisting of three phases: (1) selection of the reference parameter sets, (2) one-dimensional sensitivity analysis – determination of sensitive parameters and (3) two-dimensional sensitivity analysis – investigation of parameter surface.

#### (1) Selection of the reference parameter sets

The first phase ensures model sensitivity is tested across wide regions of parameter space rather than at a specific point. Initially, global optimisation was performed to obtain a number of parameter sets yielding a reasonable fit to the data while covering a broad region of the parameter space [1], [2]. From this larger set, a subset of reference parameter sets was chosen according to three criteria: low cost-of-fit, biologically sensible parameter values and a significant distance between the reference parameter sets. The distance was evaluated using standard techniques (*e.g.* clustering methods) and the reference parameter sets were chosen at distant locations to ensure broad coverage of parameter space. Finally, following the selection of the parameter sets from the global optimisation, local optimisation is performed on each selected set to obtain the (locally) best-fitting reference parameter sets (see also Figure 1b). The range of parameter space covered is displayed as the span of parameter values (Figure S2b).

#### (2) One-dimensional sensitivity analysis

In the second phase, for each of the locally-optimised reference parameter sets a one-dimensional sensitivity analysis was performed through stepwise alteration of each parameter across a 36-fold range of values, centred on its value in the reference parameter set. The sensitivity of the model to a particular parameter was measured through the cost-of-fit (cost function).

In the following we denote by *k*  = 1 …*N_s_* the reference parameter sets, *j*  =  1 … *N_p_* to denote the parameters within each set and *i*  =  *-N_a_* … +*N_a_* to denote the perturbation where *-* and + respectively represent negative and positive perturbations. Thus, *C_i,j,k_ (x_e_,* 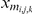 *)* is the least-square cost function (Equation 1) calculated at the *i^th^* perturbation to the *j^th^* parameter in the *k^th^* reference parameter set, where *x_e_* represents an experimental data set to be compared with its counterpart 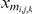 calculated through simulation of the model. The cost function is normalised within each reference parameter set with respect to its maximum computed across all parameters and perturbations, to allow meaningful comparisons among parameters despite difference in the cost-of-fit of each reference parameter set:
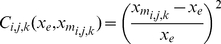
(1)

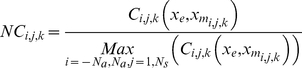
(2)


For each parameter *j* in each reference parameter set we determine two “sensitivity coefficients”: *S^size^* representing the magnitude and *S^choppy^* inferring the smoothness/variation of the calculated sensitivity.
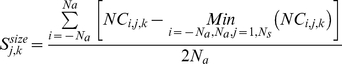
(3)

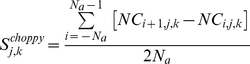
(4)


The above sensitivity coefficients are used to determine “sensitive parameters” through their means and standard deviations within each reference parameter set. For a *k^th^* parameter set, the *j^th^* parameter is subsequently defined as sensitive if
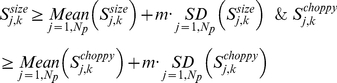
(5)where the parameter *m* indicates the strictness within which sensitivity is defined.

To determine the consistently sensitive parameters, we calculate the frequency for which a particular parameter is classified as sensitive across *N_s_* reference parameter sets. We denote by *N_j_* the number of parameter sets for which the *j^th^* parameter is classified as sensitive according to Equation (5) and define *PC_j_* as the percentage consistency for each parameter according to
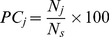
(6)


#### (3) Two-dimensional sensitivity analysis

The final phase is a two-dimensional sensitivity analysis: two of the most sensitive parameters determined by the previous analysis were chosen and perturbed simultaneously using a similar procedure of parameter perturbation and sensitivity measurement. Through variation of two parameters, we can obtain greater understanding of the surface structure of the sensitivity space via 3D plots of the cost-of-fit.

### Model Robustness Comparison

Direct and unbiased comparison of the robustness between models presents a number of challenges: models display varying complexity with respect to both topology and the number of parameters. For this study, the sensitivity between the models is compared through the *degree of robustness* (*DOR*). For each model, we compute *DOR* for whichever parameter *j* is the most sensitive within the best-fit-simulation parameter set *k*. *DOR* is defined as the inversion of the *degree of sensitivity* (*DOS*), defined as follows:
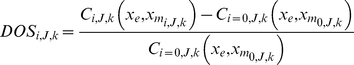
(7)where *i*  =  0 locates the zero perturbation point (at which parameter values are identical to the reference parameter set) and *J* denotes the most sensitive parameter according to Equation (5) of the parameter set *k*.

## Supporting Information

Data S1 **Model equations and parameters.** The systems of ODE equations describing one-loop and two-loop Arabidopsis circadian clock models and their corresponding best fit parameter sets.
(PDF)

Data S2 **List of experimental data for modelling Arabidopsis circadian clock.** The summary of experimental data used for matching the model simulations.
(PDF)

Figure S1 **Simulations showing fit to data for the one-loop and two-loop Arabidopsis circadian clock model using the best-fit parameter sets.**
(PDF)

Figure S2 **The characteristics of the parameters for the two-loop model used in the Consistent Robustness Analysis (CRA).**
(PDF)

Figure S3 **Simulation fit to data of the two-loop Arabidopsis circadian clock model obtained from the selected reference parameter sets (L9, L12, L13, L14, L27, and L39).**
(PDF)

Figure S4 **The sensitivity respect to parameters in the two-loop model calculated in various reference parameter sets.**
(PDF)

Figure S5 **The consistently sensitive parameters of the two-loop model identified from different criteria on sensitivity coefficients.**
(PDF)

Figure S6 **The consistently sensitive parameters of the two-loop model identified from different degree of strictness of the criteria.**
(PDF)

Figure S7 **Two-dimensional sensitivity analysis based on highly sensitive parameters for the studied reference parameter sets (L9, L12, L13, L14, L27, and L39) of the two-loop Arabidopsis circadian clock model.**
(PDF)
