# Supplementary material for: Consistent Robustness Analysis (CRA) Identifies Biologically Relevant Properties of Regulatory Network Models
Source: PLoS One. 2010 Dec 16;5(12):e15589. doi: 10.1371/journal.pone.0015589 (PMC3002950; doi:10.1371/journal.pone.0015589)
Supplement: Data S1 — Model equations and parameters. The systems of ODE equations describing one-loop and two-loop Arabidopsis circadian clock models and their corresponding best fit parameter sets. (PDF) [file pone.0015589.s001.pdf]

Model Equations and parameters

1. The one-loop model

System of ordinary differential equations for the one-loop Arabidopsis circadian clock model developed by Locke *et al* (2005) [1] are given by:

$$\frac{dc_L^{(m)}}{dt} = q_1 c_P^{(n)}\Theta(t) + \frac{n_1 c_X^{(n)^a}}{g_1 + c_X^{(n)^a}} - \frac{m_1 c_L^{(m)}}{k_1 + c_L^{(m)}} \quad \text{(S1)}$$

$$\frac{dc_L^{(c)}}{dt} = p_1 c_L^{(m)} - r_1 c_L^{(c)} + r_2 c_L^{(n)} - \frac{m_2 c_L^{(c)}}{k_2 + c_L^{(c)}} \quad \text{(S2)}$$

$$\frac{dc_L^{(n)}}{dt} = r_1 c_L^{(c)} - r_2 c_L^{(n)} - \frac{m_3 c_L^{(n)}}{k_3 + c_L^{(n)}} \quad \text{(S3)}$$

$$\frac{dc_T^{(m)}}{dt} = \frac{n_2}{g_2 + c_L^{(n)^b}} - \frac{m_4 c_T^{(m)}}{k_4 + c_T^{(m)}} \quad \text{(S4)}$$

$$\frac{dc_T^{(c)}}{dt} = p_2 c_T^{(m)} - r_3 c_T^{(c)} + r_4 c_T^{(n)} - \frac{m_5 c_T^{(c)}}{k_5 + c_T^{(c)}} \quad \text{(S5)}$$

$$\frac{dc_T^{(n)}}{dt} = r_3 c_T^{(c)} - r_4 c_T^{(n)} - \frac{m_6 c_T^{(n)}}{k_6 + c_T^{(n)}} \quad \text{(S6)}$$

$$\frac{dc_P^{(n)}}{dt} = (1 - \Theta(t))p_3 - \frac{m_7 c_P^{(n)}}{k_7 + c_P^{(n)}} - q_2\Theta(t)c_P^{(n)} \quad \text{(S7)}$$

L, *T* and *P* denote *LHY*, *TOC1* genes and P-protein while *m*, *c* and *n* indicate molecular entities and their location in the model, i.e. mRNA, protein in cytoplasm, and protein in nucleus, respectively. $\Theta(t)$ is a step function of light input to the model which set to be 1 in daytime and 0 in night-time. The parameters in Equation S7 were fixed to a constant ($p_3 = 0.5$, $q_2 = 1$ and $m_7 = k_7 = 1.2$) for all calculation. The parameters giving a good fit to the experimental data are listed in Table S1.

Table S1 Reference parameter sets for one-loop model

P-ID	P_ref	Description	L2	L26	L31	L32	L37	L41	L50
P1	*q1*	Coupling constant of light activation of LHY transcription	3.1666	1.3012	2.5364	1.4239	3.1863	2.7202	0.8757
P2	*n1*	Max. light-dependent LHY transcription rate	1.0989	21.9703	0.6138	0.8002	1.4607	2.4012	0.5103
P3	*g1*	Constant of activation by	5.536	4.7955	0.2619	21.7911	5.4946	5.9663	0.5829

		protein X							
P4	*m1*	Max. rate of LHY mRNA degradation	4.8681	1.0918	1.5846	2.256	4.9998	4.1488	0.5549
P5	*k1*	Michaelis constant of LHY mRNA degradation	4.6555	1.3569	1.185	2.7057	10.909	0.7151	0.3175
P6	*p1*	Rate constant of LHY mRNA translation	2.3546	0.4406	1.3504	14.5907	1.8309	3.3273	1.888
P7	*r1*	Rate constant of LHY transport into nucleus	5.6125	1.2781	0.267	0.6619	1.5	8.4697	15.5308
P8	*r2*	Rate constant of LHY transport out of nucleus	10.2606	0.7005	0.0724	1.0381	1.6581	3.0762	0.5855
P9	*m2*	Max. rate of cytoplasmic LHY degradation	1.9998	0.449	0.1265	0.3448	2.6183	9.0808	0.385
P10	*k2*	Michaelis constant of cytoplasmic LHY degradation	3.3853	0.1721	144.4148	1.8125	0.9945	2.8632	0.0116
P11	*m3*	Max. rate of nuclear LHY degradation	1.6496	0.0635	0.7823	2.9233	0.4034	1.2364	0.462
P12	*k3*	Michaelis constant of nuclear LHY degradation	1.3424	0.3455	0.0378	0.0428	0.1175	3.9305	0.3389
P13	*n2*	Max. TOC1 transcription rate	1.0304	0.1024	0.363	9.9479	7.6157	4.4108	8.3177
P14	*g2*	Constant of activation by TOC1	1.4531	0.0101	0.2506	3.6589	0.7033	1.1192	1.0426
P15	*m4*	Max. rate of TOC1 mRNA degradation	3.8873	13.854	2.2049	30.6524	15.502	4.6163	9.1262
P16	*k4*	Michaelis constant of TOC1 mRNA degradation	6.8398	0.7804	0.3991	3.9875	0.6635	8.4728	0.14
P17	*p2*	Rate constant of TOC1 mRNA translation	7.4944	0.7897	0.7256	8.0792	2.2714	0.5303	0.629
P18	*r3*	Rate constant of TOC1 transport into nucleus	3.1687	0.0036	2.0362	4.9074	0.0537	5.4225	0.1808
P19	*r4*	Rate constant of TOC1 transport out of nucleus	1.4383	0.1974	0.0023	0.682	0.0161	2.7472	0.0018

P20	*m5*	Max. rate of cytoplasmic TOC1 degradation	6.2541	1.0602	0.1	0.9868	2.7194	4.2634	0.3836
P21	*k5*	Michaelis constant of cytoplasmic TOC1 degradation	0.0829	0.0712	216.756	0.4102	10.3796	0.1124	0.5003
P22	*m6*	Max. rate of nuclear TOC1 degradation	0.0275	0.0013	0.4586	1.8897	1.1916	2.076	0.1836
P23	*k6*	Michaelis constant of nuclear TOC1 degradation	0.4506	2.8329	0.0075	0.1485	0.476	0.3477	0.0048
P24	*a*	Hill coefficient of repression by protein LHY	2.174	2.0819	1.6336	1.7532	2	2	2.0407
P25	*b*	Hill coefficient of activation by protein TOC1	0.8868	1.0142	1.0152	0.9489	1	1	0.8571

2. The two-loop model

Two-loop model was later extended from one-loop model by Locke *et al* (2005) [2] which can be described as followed:

$$\frac{dc_L^{(m)}}{dt} = q_1 c_P^{(n)}\Theta(t) + \frac{n_1 c_X^{(n)^a}}{g_1 + c_X^{(n)^a}} - \frac{m_1 c_L^{(m)}}{k_1 + c_L^{(m)}} \quad \text{(S8)}$$

$$\frac{dc_L^{(c)}}{dt} = p_1 c_L^{(m)} - r_1 c_L^{(c)} + r_2 c_L^{(n)} - \frac{m_2 c_L^{(c)}}{k_2 + c_L^{(c)}} \quad \text{(S9)}$$

$$\frac{dc_L^{(n)}}{dt} = r_1 c_L^{(c)} - r_2 c_L^{(n)} - \frac{m_3 c_L^{(n)}}{k_3 + c_L^{(n)}} \quad \text{(S10)}$$

$$\frac{dc_T^{(m)}}{dt} = \left(\frac{n_2 c_Y^{(n)^b}}{g_2 + c_Y^{(n)^b}}\right)\left(\frac{n_3}{g_3 + c_L^{(n)^b}}\right) - \frac{m_4 c_T^{(m)}}{k_4 + c_T^{(m)}} \quad \text{(S11)}$$

$$\frac{dc_T^{(c)}}{dt} = p_2 c_T^{(m)} - r_3 c_T^{(c)} + r_4 c_T^{(n)} - \left(\left(1-\Theta(t)\right)m_5 + m_6\right)\frac{c_T^{(c)}}{k_5 + c_T^{(c)}} \quad \text{(S12)}$$

$$\frac{dc_T^{(n)}}{dt} = r_3 c_T^{(c)} - r_4 c_T^{(n)} - \left(\left(1-\Theta(t)\right)m_7 + m_8\right)\frac{c_T^{(n)}}{k_6 + c_T^{(n)}} \quad \text{(S13)}$$

$$\frac{dc_X^{(m)}}{dt} = \frac{n_4 c_T^{(n)^c}}{g_4 + c_X^{(n)^c}} - \frac{m_9 c_X^{(m)}}{k_7 + c_X^{(m)}} \quad \text{(S14)}$$

$$\frac{dc_X^{(c)}}{dt} = p_3 c_X^{(m)} - r_5 c_X^{(c)} + r_6 c_X^{(n)} - \frac{m_{10} c_X^{(c)}}{k_8 + c_X^{(c)}} \quad \text{(S15)}$$

$$\frac{dc_X^{(n)}}{dt} = r_5 c_X^{(c)} - r_6 c_X^{(n)} - \frac{m_{11} c_X^{(n)}}{k_{93} + c_X^{(n)}} \quad \text{(S16)}$$

$$\frac{dc_Y^{(m)}}{dt} = \left(\Theta(t) q_2 c_P^{(n)} + \frac{(\Theta(t) n_5 + n_6)}{g_5 + c_T^{(n)d}} \right) \left(\frac{n_7}{g_6 + c_L^{(n)e}} \right) - \frac{m_{12} c_Y^{(m)}}{k_{10} + c_Y^{(m)}} \quad \text{(S17)}$$

$$\frac{dc_Y^{(c)}}{dt} = p_4 c_Y^{(m)} - r_7 c_Y^{(c)} + r_8 c_Y^{(n)} - \frac{m_{13} c_Y^{(c)}}{k_{11} + c_Y^{(c)}} \quad \text{(S18)}$$

$$\frac{dc_Y^{(n)}}{dt} = r_7 c_Y^{(c)} - r_8 c_Y^{(n)} - \frac{m_{14} c_Y^{(n)}}{k_{12} + c_Y^{(n)}} \quad \text{(S19)}$$

$$\frac{dc_P^{(n)}}{dt} = (1 - \Theta(t)) p_5 - \frac{m_{15} c_P^{(n)}}{k_{13} + c_P^{(n)}} - q_3 \Theta(t) c_P^{(n)} \quad \text{(S20)}$$

The additional variable denoted *X* and *Y* represent hypothetical genes *X* and *Y*, respectively. In the similar manner to one-loop model, the kinetics of P-protein explained in Equation S20 were fixed ($p_5 = 0.5$, $q_3 = 1$ and $m_{15} = k_{13} = 1.2$) for all calculation. A group of best fit parameter sets are listed in Table S2.

Table S2 Reference parameter sets for two-loop model

P-ID	P_ref	Description	L0	L9	L12	L13	L14	L27	L39
P1	*q1*	Coupling constant of light activation of LHY transcription	2.5759	3.8413	2.5367	1.7231	2.2018	1.2087	3.7329
P2	*n1*	Max. light-dependent *LHY* transcription rate	5.1495	1.6058	0.6792	3.2804	0.8771	0.599	3.5951
P3	*g1*	Constant of activation by protein X	0.6717	0.2428	1.3939	7.4018	0.2504	0.6314	0.1009
P4	*m1*	Max. rate of *LHY* mRNA degradation	1.5912	3.0014	2.0259	5.0267	2.0428	1.3876	16.1289
P5	*k1*	Michaelis constant of *LHY* mRNA degradation	1.8528	1.9837	2.7764	5.0965	4.9709	0.9156	4.9657
P6	*p1*	Rate constant of *LHY* mRNA translation	0.8128	0.1885	44.5794	1.7025	1.5149	4.0477	0.6933
P7	*r1*	Rate constant of LHY transport into nucleus	17.2388	77.0779	0.8139	4.9858	6.6294	0.1715	8.6249
P8	*r2*	Rate constant of LHY transport out of nucleus	0.1778	1.5279	0.5301	1.0864	0.9658	1.539	16.5107
P9	*m2*	Max. rate of cytoplasmic LHY degradation	21.1058	67.0401	77.0427	128.7237	1.3078	3.6355	5.5115

P10	*k2*	Michaelis constant of cytoplasmic LHY degradation	1.6292	7.8909	3.131	17.4919	1.0333	16.9954	4.2174
P11	*m3*	Max. rate of nuclear LHY degradation	3.5018	0.399	1.0242	1.9527	8.2634	0.1083	1.3385
P12	*k3*	Michaelis constant of nuclear LHY degradation	1.2565	1.0606	0.2078	2.3396	1.5385	1.1721	1.605
P13	*n2*	Max. *TOC1* transcription rate	1.4233	0.4302	3.8828	3.6335	1.7983	2.6984	1.8479
P14	*g2*	Constant of activation by protein Y	0.0333	2.7987	0.2446	2.3172	0.0192	2.6659	7.8532
P15	*n3*	Constant regulation by protein Y	0.5251	21.5704	2.041	3.5802	8.7261	8.5338	0.1641
P16	*g3*	Constant of repression by protein LHY	0.266	0.1516	0.0706	0.2275	0.1876	3.5819	0.0179
P17	*m4*	Max. rate of *TOC1* mRNA degradation	3.9748	8.5773	15.923	2.4186	108.4974	7.8459	0.7966
P18	*k4*	Michaelis constant of *TOC1* mRNA degradation	2.4823	12.2813	6.6023	2.1043	102.0495	3.431	4.7869
P19	*p2*	Rate constant of *TOC1* mRNA translation	4.2228	0.1273	0.5086	0.1675	0.1692	4.8662	0.3081
P20	*r3*	Rate constant of TOC1 transport into nucleus	0.323	8.3445	9.5851	0.994	0.1696	1.4029	0.7487
P21	*r4*	Rate constant of TOC1 transport out of nucleus	1.9641	0.4903	3.1533	0.4668	0.2308	2.7209	0.1818
P22	*m5*	Max. rate of light dependent cytoplasmic TOC1 degradation	0.0012	0.7468	0.0018	1.5405	0.0225	0.8928	0.0133
P23	*m6*	Max. rate of light independent cytoplasmic TOC1 degradation	3.2372	2.3301	0.0368	1.4918	4.5318	2.2566	0.2589
P24	*k5*	Michaelis constant of cytoplasmic TOC1 degradation	2.9809	6.2478	6.1054	9.2669	0.686	1.1228	2.5309
P25	*m7*	Max. rate of light dependent nuclear TOC1 degradation	0.0521	0.1789	0.0147	1.3708	0.009	1.12	0.3794
P26	*m8*	Max. rate of light independent nuclear TOC1 degradation	3.897	1.7547	11.4511	22.0606	3.0372	4.7235	6.7745
P27	*k6*	Michaelis constant of nuclear TOC1 degradation	0.3722	4.2643	0.8095	2.3301	0.6446	6.2498	6.9043

P28	*n4*	Max. *X* transcription rate	0.2213	0.305	0.4354	1.9754	0.2373	1.1581	0.1162
P29	*g4*	Constant of activation by protein TOC1	0.4181	0.6339	0.1051	1.4192	0.0057	4.3917	0.1566
P30	*m9*	Max. rate of *X* mRNA degradation	9.9471	0.8853	0.4175	2.6405	0.3617	2.8288	1.1757
P31	*k7*	Michaelis constant of *X* mRNA degradation	6.5903	1.6147	1.6033	0.3191	1.8639	2.9283	4.9187
P32	*p3*	Rate constant of *X* mRNA translation	2.1317	7.4982	1.4074	10.6624	0.3469	5.395	0.3045
P33	*r5*	Rate constant of protein X transport into nucleus	1.0439	1.3821	2.3817	1.2937	1.3362	9.2475	0.8694
P34	*r6*	Rate constant of protein X transport out of nucleus	3.3344	0.7193	18.7256	0.5752	3.2545	1.9791	0.4407
P35	*m10*	Max. rate of degradation of cytoplasmic protein X	0.2069	0.2177	0.4077	0.0779	1.2357	4.7809	0.4554
P36	*k8*	Michaelis constant of cytoplasmic protein X degradation	0.6613	0.1848	0.1237	1.1925	3.4668	0.1466	0.5613
P37	*m11*	Max. rate of degradation of nuclear protein X	3.3455	5.2661	4.1432	0.0065	0.0397	1.6864	0.0323
P38	*k9*	Michaelis constant of nuclear protein X degradation	17.9958	1.5912	2.5313	0.5228	0.4122	0.8041	2.1029
P39	*n5*	Light dependent component of *Y* transcription	1.9755	0.0257	0.2275	0.3728	0.0228	0.1646	0.0344
P40	*n6*	Light independent component of *Y* transcription	3.4709	0.4373	4.546	4.0605	1.4893	4.6144	0.7489
P41	*g5*	Constant of repression by TOC1	1.7594	1.2635	0.026	0.0466	0.2168	4.5496	0.1729
P42	*m12*	Max. rate of *Y* mRNA degradation	4.1362	0.5178	0.8096	3.1375	2.1834	1.2943	0.4397
P43	*k10*	Michaelis constant of *Y* mRNA degradation	1.7396	0.6616	1.9072	5.4739	2.2283	1.5324	0.1276
P44	*p4*	Rate constant of *Y* mRNA translation	0.266	1.7086	0.0116	0.4371	0.0095	0.3691	1.34
P45	*r7*	Rate constant of protein Y transport into nucleus	2.0966	3.527	11.3545	7.3215	7.8454	4.6565	5.773
P46	*r8*	Rate constant of protein Y transport out of nucleus	0.1866	0.5981	0.4384	2.035	0.0612	0.7638	0.166

P47	*m13*	Max. rate of degradation of cytoplasmic protein Y	0.1195	1.3338	1.6329	3.3043	0.0036	1.164	0.1117
P48	*k11*	Michaelis constant of cytoplasmic protein Y degradation	1.9429	0.3107	0.1667	0.8502	1.4187	5.9689	8.082
P49	*m14*	Max. rate of degradation of nuclear protein Y	0.5921	0.0401	0.4615	0.5603	0.148	0.2348	5.3312
P50	*k12*	Michaelis constant of nuclear protein Y degradation	1.7554	0.7602	0.2867	7.8684	0.4106	4.5515	5.421
P51	*n7*	Constant regulation by protein LHY	0.0052	0.4094	0.1353	0.0359	0.06	3.9315	0.2572
P52	*g6*	Constant of repression by LHY	0.0613	1.1662	19.6203	2.3461	0.9981	10.6559	2.6725
P53	*q2*	Coupling constant of light activation of *Y* transcription	28.4839	4.6552	121.059	112.8891	99.3433	3.7924	13.3767
P54	*b*	Hill coefficient of activation by protein Y	1.0681	1.1896	1.0711	2.8985	1.0281	2.277	1.1647
P55	*a*	Hill coefficient of activation by protein X	3.144	3.7638	3.9613	3.1829	3.8861	3.7535	2.3391
P56	*c*	Hill coefficient of activation by protein TOC1	1.4672	2.188	1.6784	1.5472	3.5827	4	1.7748
P57	*e*	Hill coefficient of repression by protein LHY for *Y*	1.0588	1	1.8493	1.499	1.7576	1.0095	1.6571
P58	*d*	Hill coefficient of activation by protein TOC1 for *Y*	3.5161	2.5409	4	1.5366	1.8123	3.4889	3.8342

References

1. Locke JCW, Millar, A. J., Turner M. S. : **Modelling genetic networkswith noisy and varied experimental data: the circadian clock in *Arabidopsis thaliana*.** *Journal of Theoretical Biology* 2005, **234:**383-393.
2. Locke JCW, Southern, M. M., Kozma-Bognar, L., Hibberd, V., Brown, P. E., Turner, M. S., Millar, A. J.: **Extension of a genetic network model by iterative experimentation and mathematical analysis.** *Molecular Systems Biology* 2005.
