# Supplementary material for: Consistent Robustness Analysis (CRA) Identifies Biologically Relevant Properties of Regulatory Network Models
Source: PLoS One. 2010 Dec 16;5(12):e15589. doi: 10.1371/journal.pone.0015589 (PMC3002950; doi:10.1371/journal.pone.0015589)
Supplement: Data S2 — List of experimental data for modelling Arabidopsis circadian clock. The summary of experimental data used for matching the model simulations. (PDF) [file pone.0015589.s002.pdf]

Table S3 The experimental data for Arabidopsis circadian clock modelling

Backgrounds	Genes	Photoperiods*	References
wild-type	*LHY/CCA1*	LL	Mizoguchi *et al*. (2002) Developmental Cell, 2, 629-641 Kim *et al*. (2003) The EMBO Journal, 22 (4), 935-944
		16L8D	Mizoguchi *et al*. (2002) Developmental Cell, 2, 629-641
		8L16D	Roden *et al*. (2001) Cell & Developmental Biology, 12, 305-315
		DD	Salome *et al.* (2005) The Plant Cell, 17, 791-803
	TOC1	LL	Mizoguchi *et al*. (2002) Developmental Cell, 2, 629-641
		16L8D	Mizoguchi *et al*. (2002) Developmental Cell, 2, 629-641
		8L16D	Edwards K. unpublished data
		DD	Salome *et al.* (2005) The Plant Cell, 17, 791-803
	GI	LL	Mizoguchi *et al*. (2002) Developmental Cell, 2, 629-641
		16L8D	Mizoguchi *et al*. (2002) Developmental Cell, 2, 629-641
lhycca1 double mutant	*TOC1*	LL	Mizoguchi *et al*. (2002) Developmental Cell, 2, 629-641
	GI	LL	Mizoguchi *et al*. (2002) Developmental Cell, 2, 629-641

* Notes: LL – constant light, 16L8D – 16 hours of light and 8 hours of darkness, 8L16D – 8 hours of light and 16 hours of darkness, and DD – constant darkness.
