# Supplementary material for: Consistent Robustness Analysis (CRA) Identifies Biologically Relevant Properties of Regulatory Network Models
Source: PLoS One. 2010 Dec 16;5(12):e15589. doi: 10.1371/journal.pone.0015589 (PMC3002950; doi:10.1371/journal.pone.0015589)

Figure S1 The model circuits with the best fit results to time-series RNA expression from (a) one-loop model using *L26* and (b) two-loop model using *L0*. The blue solid line with marker demonstrates the experimental data while the pink and red solid line indicates the simulated results of the model. Here, three conditions were selected from many simulations to present the goodness-of-fit of the models, comprising of *TOC1* expression in wild-type under constant light (WT-LL) and 16:8 light;dark cycle (WT-16L8D), and *TOC1* expression in *lhycca1* double mutant under constant light (*lhycca*-LL) The blue solid line with marker demonstrates the experimental data while the pink and red solid line indicates the model simulated results. The y-axis is the RNA concentration, while the x-axis is time in hour. Note that unlike the fitted result for the two-loop model, the simulated *TOC1* expression in *lhycca1* double mutant is a predicted result for the one-loop model,.

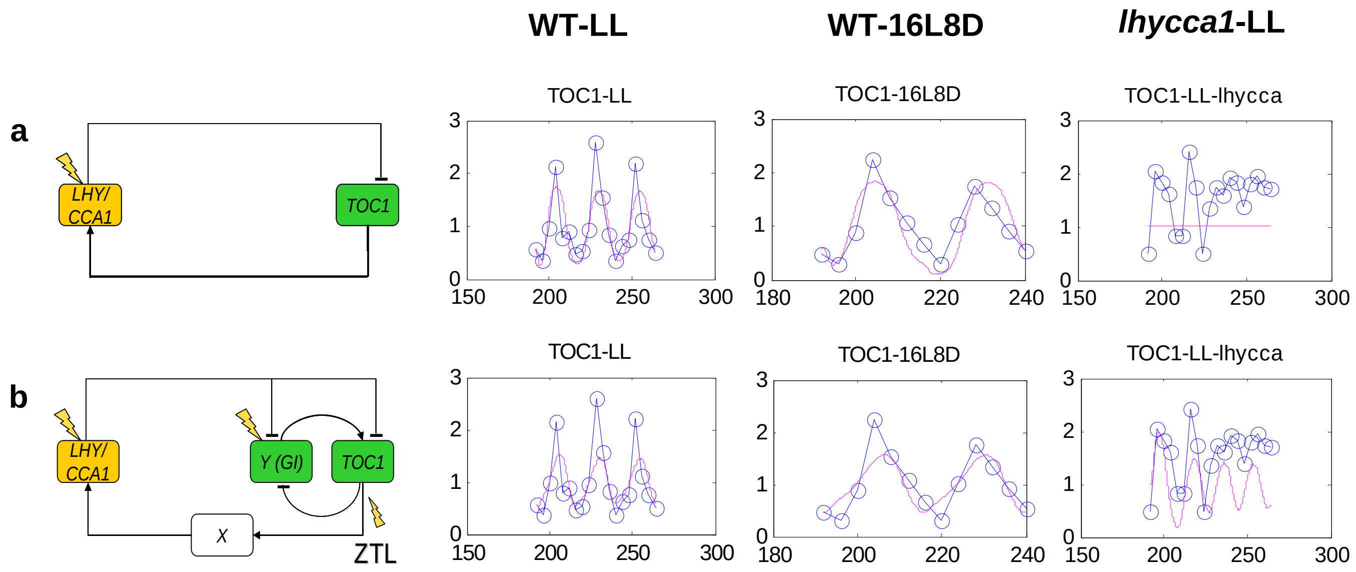

Supplement: Figure S1 — Simulations showing fit to data for the one-loop and two-loop Arabidopsis circadian clock model using the best-fit parameter sets. (PDF) [file pone.0015589.s003.pdf]
