# Supplementary material for: Consistent Robustness Analysis (CRA) Identifies Biologically Relevant Properties of Regulatory Network Models
Source: PLoS One. 2010 Dec 16;5(12):e15589. doi: 10.1371/journal.pone.0015589 (PMC3002950; doi:10.1371/journal.pone.0015589)

Figure S2 Parameter characteristics. (a) The hierarchical clustering result of 50 parameter sets of the two-loop model obtained from global optimisation which displays the relative distance between the parameter sets on the parameter space. The green circle marks the *set0* and the red asterisk depicts the other selected reference parameter sets used along the sensitivity analysis of two-loop model. (b) The parameter span of reasonable behaviour in the two-loop model represented from seven re-optimised reference parameter sets (*Lxx*, the re-optimisation of the selected parameter sets marked with red asterisks in (a) (*set xx*)) indicates the wide boundary of analyzed parameter space. Note that the parameters P54 to P58 shown in (b) represent Hill coefficients used in the two-loop model, the values of which are constrained within a range of 1 to 4.

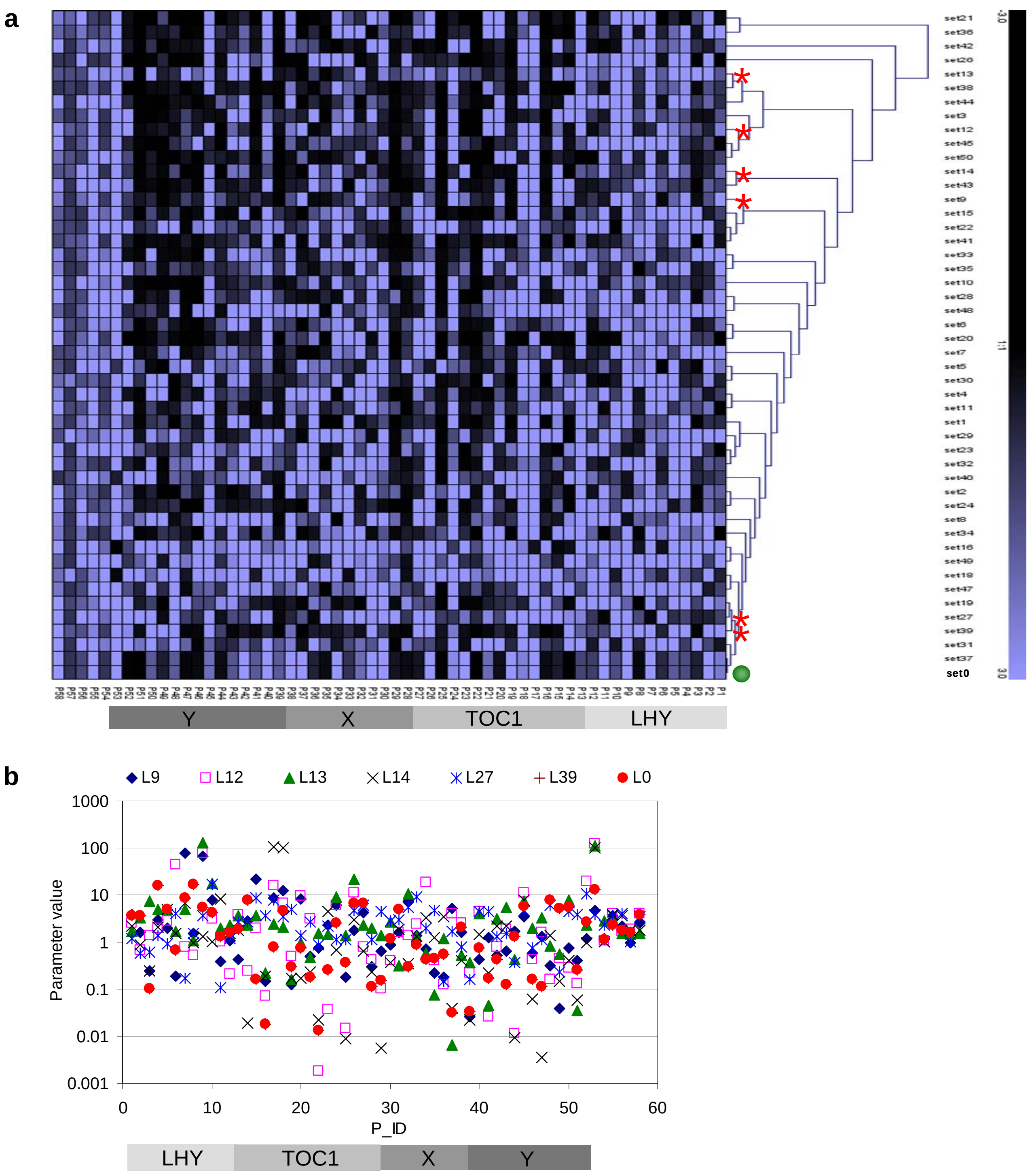

Supplement: Figure S2 — The characteristics of the parameters for the two-loop model used in the Consistent Robustness Analysis (CRA). (PDF) [file pone.0015589.s004.pdf]
