# Supplementary material for: Consistent Robustness Analysis (CRA) Identifies Biologically Relevant Properties of Regulatory Network Models
Source: PLoS One. 2010 Dec 16;5(12):e15589. doi: 10.1371/journal.pone.0015589 (PMC3002950; doi:10.1371/journal.pone.0015589)

Figure S3 The fit results of RNA expression time-series from the two-loop model using re-optimised parameter sets: *L9*, *L12*, *L13*, *L14*, *L27* and *L39* (the re-optimised parameter sets of *set9*, *set 12*, *set13*, *set14*, *set27* and *set39*, respectively). Here, three selected simulations of all twelve fitted RNA time-series were presented, comprising of *TOC1* expression in wild-type under constant light (WT-LL) and 16:8 light;dark cycle (WT-16L8D), and *TOC1* expression in *lhycca1* double mutant under constant light (*lhycca*-LL) The blue solid line with marker demonstrates the experimental data while the pink and red solid line indicates the model simulated results. The y-axis is the RNA concentration, while the x-axis is time in hour.

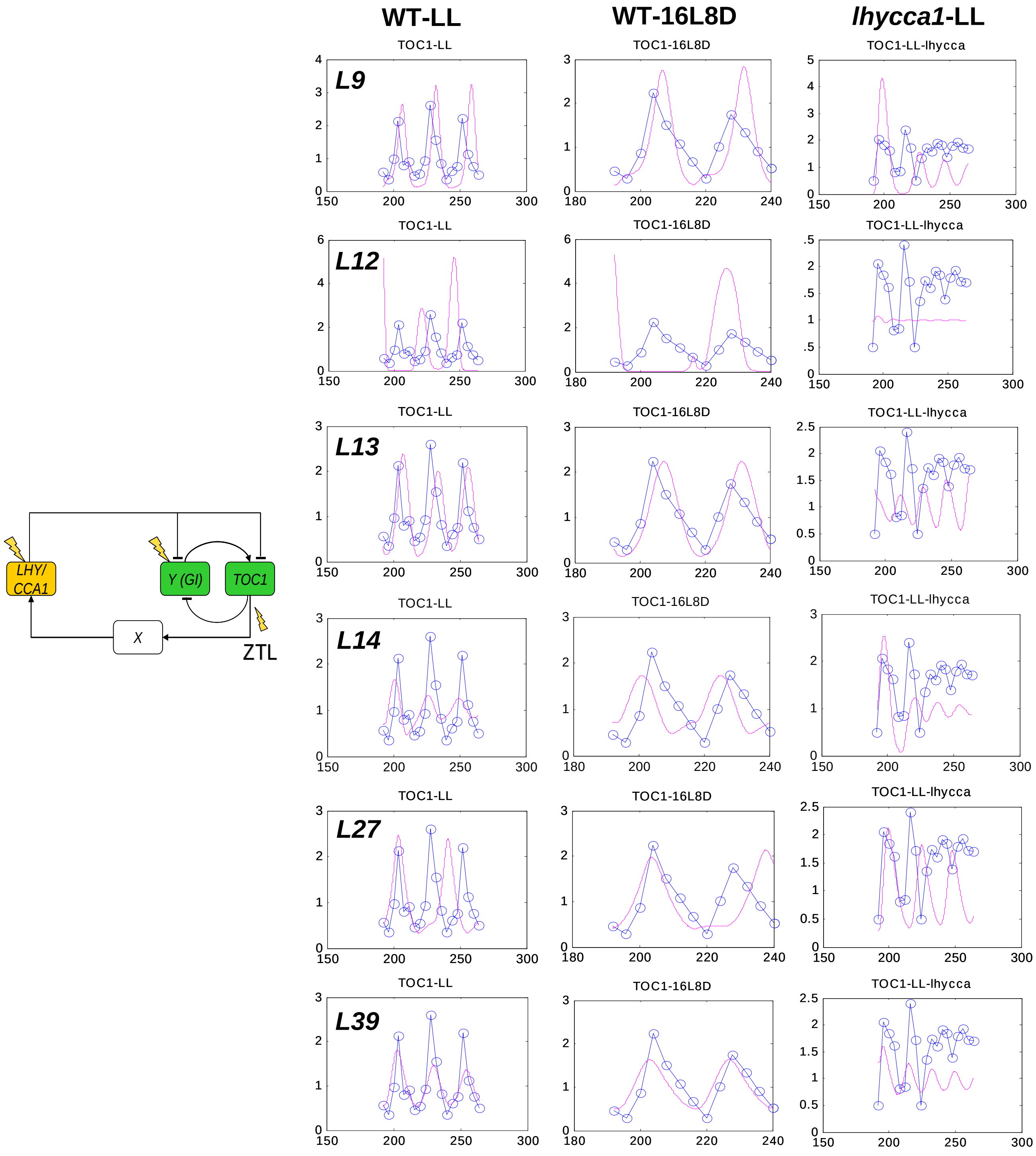

Supplement: Figure S3 — Simulation fit to data of the two-loop Arabidopsis circadian clock model obtained from the selected reference parameter sets (L9, L12, L13, L14, L27, and L39). (PDF) [file pone.0015589.s005.pdf]
