# Supplementary material for: Consistent Robustness Analysis (CRA) Identifies Biologically Relevant Properties of Regulatory Network Models
Source: PLoS One. 2010 Dec 16;5(12):e15589. doi: 10.1371/journal.pone.0015589 (PMC3002950; doi:10.1371/journal.pone.0015589)

Figure S4 The sensitivity respect to parameters in the two-loop model calculated in various parameter sets: (a) *L9* (b) *L12* (c) *L13* (d) *L14* (e) *L27* (f) *L39*. The heatmap plots the sensitivity and robustness of the model at any parameters (row) and perturbations (column).

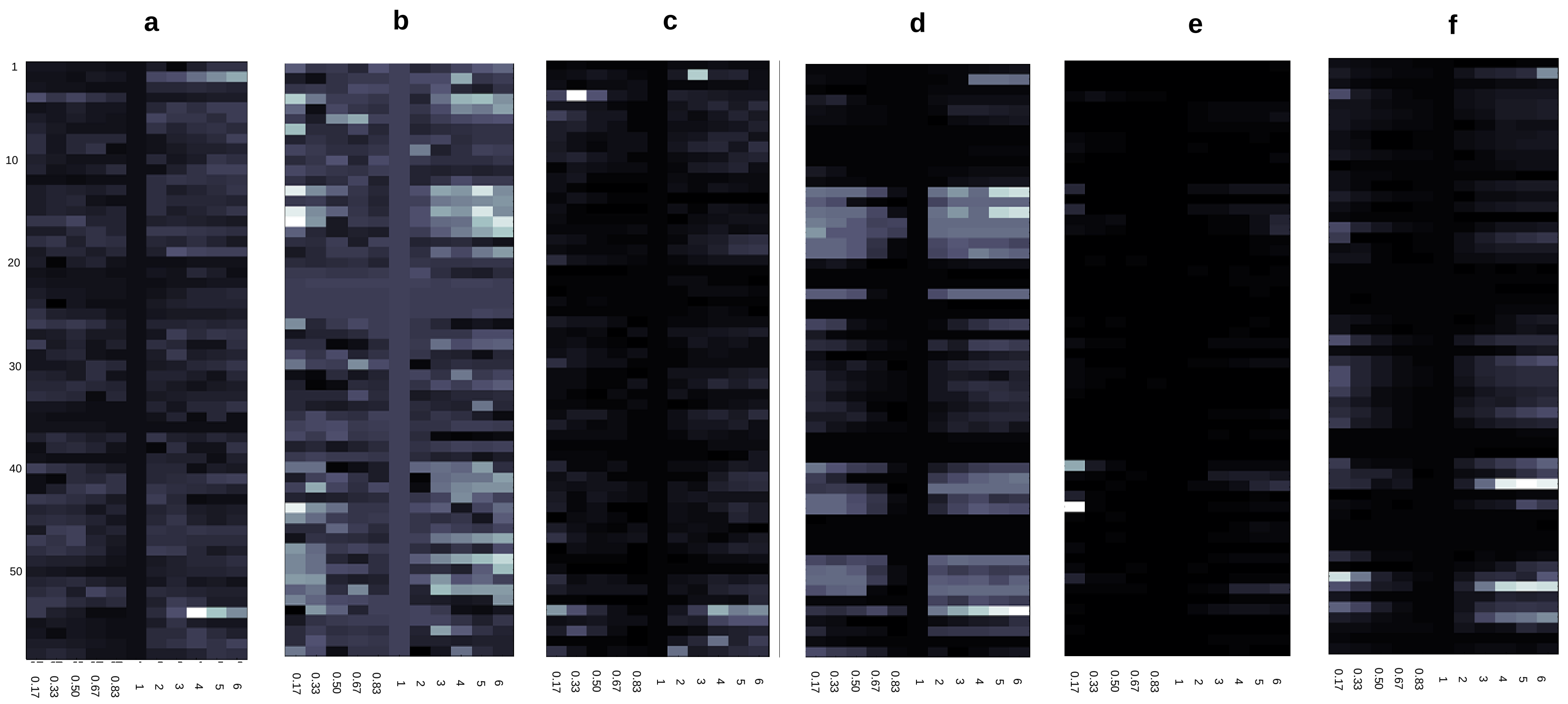

Supplement: Figure S4 — The sensitivity respect to parameters in the two-loop model calculated in various reference parameter sets. (PDF) [file pone.0015589.s006.pdf]
