# Supplementary material for: Consistent Robustness Analysis (CRA) Identifies Biologically Relevant Properties of Regulatory Network Models
Source: PLoS One. 2010 Dec 16;5(12):e15589. doi: 10.1371/journal.pone.0015589 (PMC3002950; doi:10.1371/journal.pone.0015589)

Figure S5 The percent consistency (PC) of the sensitive parameters among the reference parameter sets calculated based on the different criteria of sensitivity coefficients was plotted according to the genes (a) and molecular processes (b) (TL= translation and T = Transportation)

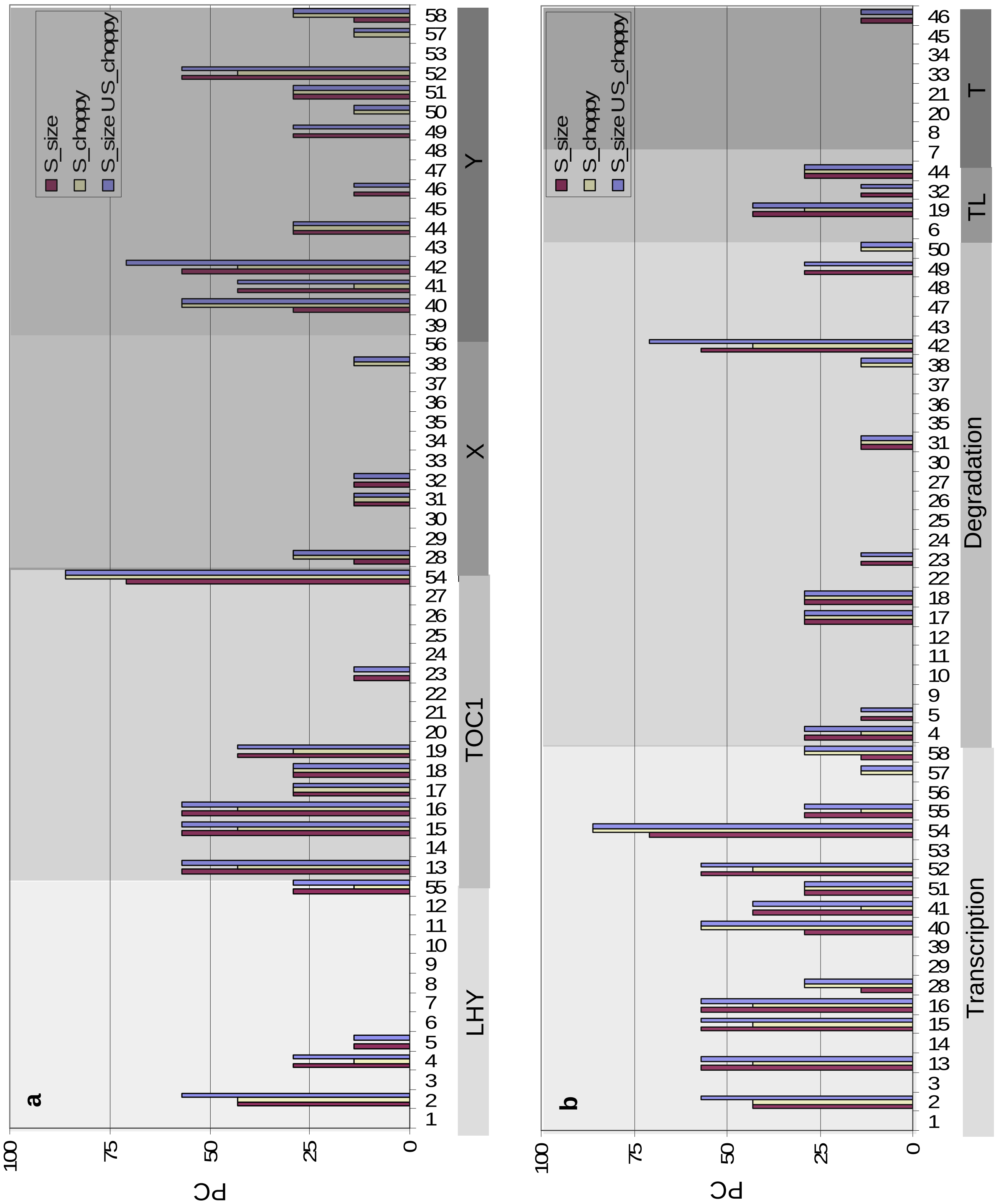

Supplement: Figure S5 — The consistently sensitive parameters of the two-loop model identified from different criteria on sensitivity coefficients. (PDF) [file pone.0015589.s007.pdf]
