# Supplementary material for: Consistent Robustness Analysis (CRA) Identifies Biologically Relevant Properties of Regulatory Network Models
Source: PLoS One. 2010 Dec 16;5(12):e15589. doi: 10.1371/journal.pone.0015589 (PMC3002950; doi:10.1371/journal.pone.0015589)

Figure S6 The percent consistency (PC) of the parameters obtained from different degree of strictness (0.5SD (m = 0.5) (a) and 2SD (m = 2) (b)) to classify the sensitive parameters (based on S^{size} and S^{choppy}) was plotted according to the (1) genes and molecular processes (2) (TL= translation and T = Transportation). The consistently sensitive parameters marked as black bars were classified based on 50 percent consistency cut-off.

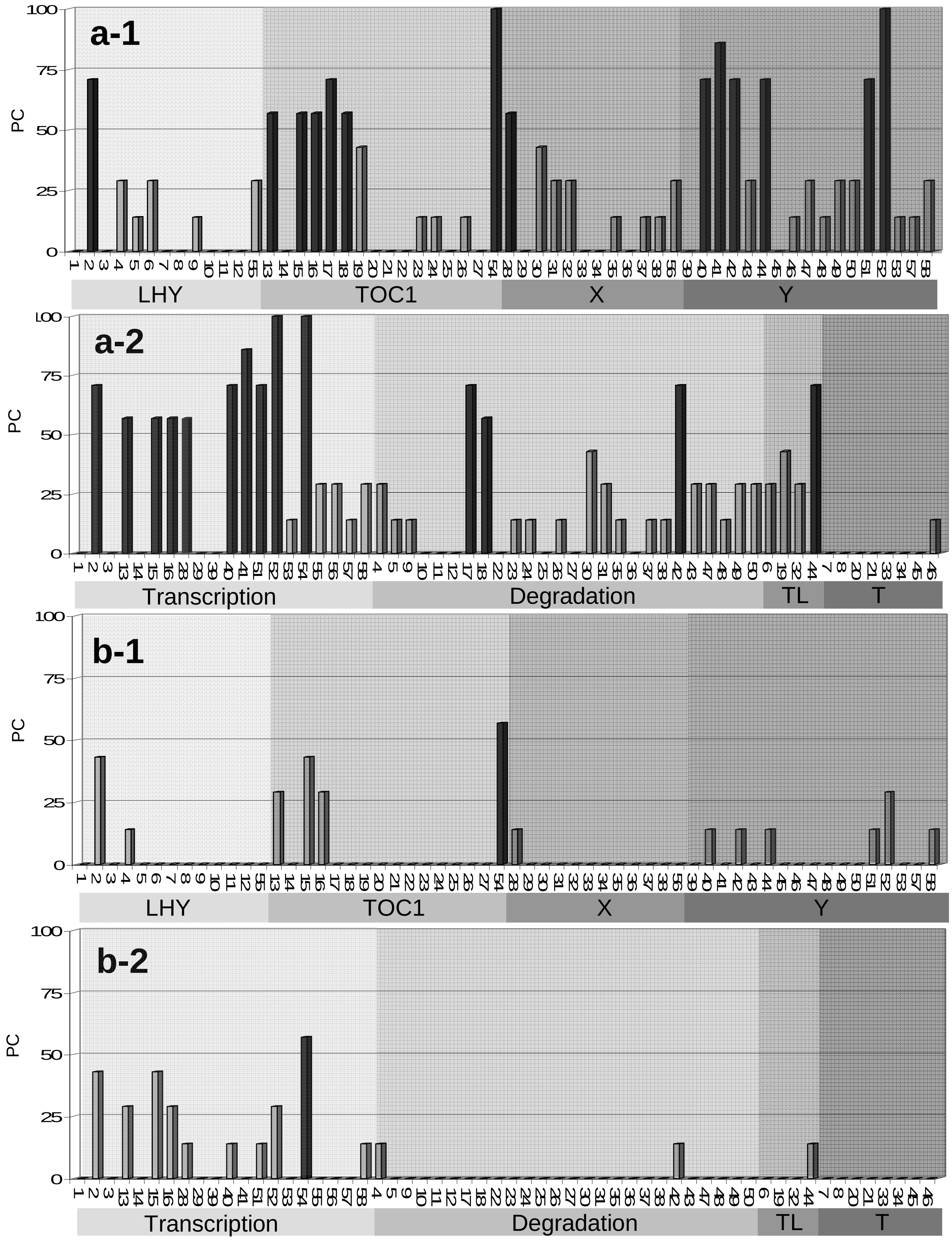

Supplement: Figure S6 — The consistently sensitive parameters of the two-loop model identified from different degree of strictness of the criteria. (PDF) [file pone.0015589.s008.pdf]
