# Supplementary material for: Consistent Robustness Analysis (CRA) Identifies Biologically Relevant Properties of Regulatory Network Models
Source: PLoS One. 2010 Dec 16;5(12):e15589. doi: 10.1371/journal.pone.0015589 (PMC3002950; doi:10.1371/journal.pone.0015589)

Figure S7 The parameter surface developed from 2D sensitivity analysis of the two-loop model based on the highly sensitive parameters of each reference parameter set (*L9*, *L12*, *L13*, *L14*, *L27*, and *L39)*: P2 (*n1*)-LHY transcription, P4 (*m1*)-Max. *LHY* mRNA degradation rate, P13 (*n2*)-Max. *TOC1* transcription rate, P16 (*g3*)-*TOC1* transcription, P19 (*p2*)-Rate constant of *TOC1* mRNA translation, P42 (*m12*)-*Y* mRNA degradation rate, P49 (*m14*)-Yp in cytosol degradation, P52 (*g6*)-*Y* transcription rate, and P54 (*b*)-Hill coefficient of activation by protein Y. The red star illustrates the position of reference parameter set which is always the minimum cost region on the parameter surface. X and Y axis are the perturbation of sensitive parameters and Z axis is cost function corresponding to the parameter perturbation.

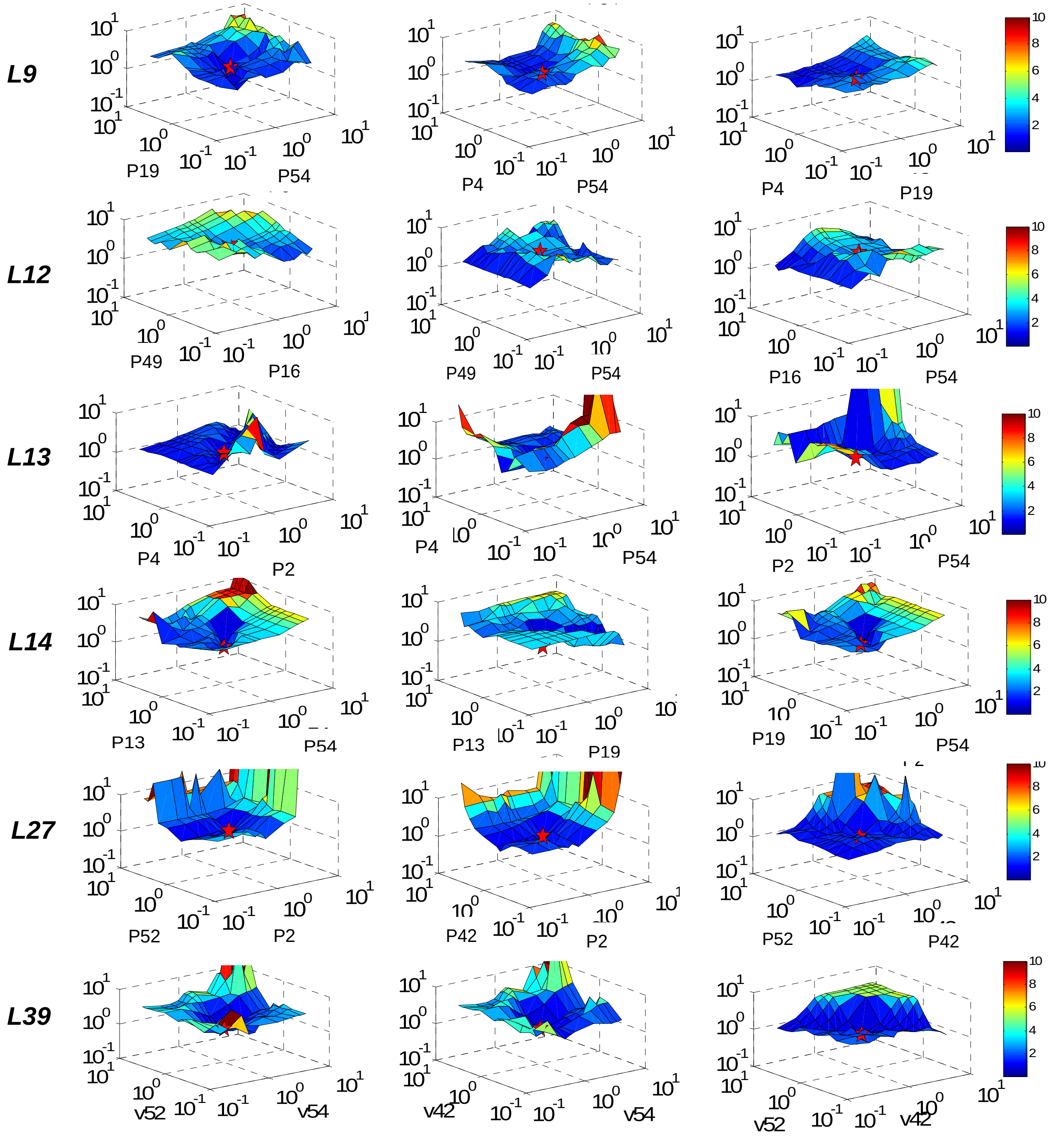

Supplement: Figure S7 — Two-dimensional sensitivity analysis based on highly sensitive parameters for the studied reference parameter sets (L9, L12, L13, L14, L27, and L39) of the two-loop Arabidopsis circadian clock model. (PDF) [file pone.0015589.s009.pdf]
